# Adhesion of *Plasmodium falciparum* infected erythrocytes in ex vivo perfused placental tissue: a novel model of placental malaria

**DOI:** 10.1186/s12936-016-1342-2

**Published:** 2016-05-26

**Authors:** Caroline Pehrson, Line Mathiesen, Kristine K. Heno, Ali Salanti, Mafalda Resende, Ron Dzikowski, Peter Damm, Stefan R. Hansson, Christopher L. King, Henning Schneider, Christian W. Wang, Thomas Lavstsen, Thor G. Theander, Lisbeth E. Knudsen, Morten A. Nielsen

**Affiliations:** Centre for Medical Parasitology at Department of Immunology and Microbiology, Faculty of Health and Medical Sciences, University of Copenhagen and at Department of Infectious Diseases, Copenhagen University Hospital (Rigshospitalet), Copenhagen, Denmark; Section of Environmental Health, Department of Public Health, University of Copenhagen, Øster Farimagsgade 5A, 1353 Copenhagen, Denmark; Department of Microbiology and Molecular Genetics, The Institute for Medical Research Israel-Canada, The Kuvin Center for the Study of Infectious and Tropical Diseases, The Hebrew University-Hadassah Medical School, 91120 Jerusalem, Israel; Department of Obstetrics, Rigshospitalet, Faculty of Health and Medical Sciences, University of Copenhagen, Blegdamsvej 9, 2100 Copenhagen Ø, Denmark; Division of Obstetrics and Gynecology, Department of Clinical Sciences Lund, Lund University, Lund, Sweden; Center for Global Health and Diseases, Case Western Reserve University and Veterans Affairs Medical Center, Cleveland, USA; Department of Obstetrics and Gynecology, Inselspital, Bern University Hospital, University of Bern, Bern, Switzerland

**Keywords:** Placental malaria, Placental perfusion, VAR2CSA

## Abstract

**Background:**

Placental malaria occurs when *Plasmodium falciparum* infected erythrocytes sequester in the placenta. Placental parasite isolates bind to chondroitin sulphate A (CSA) by expression of VAR2CSA on the surface of infected erythrocytes, but may sequester by other VAR2CSA mediated mechanisms, such as binding to immunoglobulins. Furthermore, other parasite antigens have been associated with placental malaria. These findings have important implications for placental malaria vaccine design. The objective of this study was to adapt and describe a biologically relevant model of parasite adhesion in intact placental tissue.

**Results:**

The ex vivo placental perfusion model was modified to study adhesion of infected erythrocytes binding to CSA, endothelial protein C receptor (EPCR) or a transgenic parasite where *P. falciparum* erythrocyte membrane protein 1 expression had been shut down. Infected erythrocytes expressing VAR2CSA accumulated in perfused placental tissue whereas the EPCR binding and the transgenic parasite did not. Soluble CSA and antibodies specific against VAR2CSA inhibited binding of infected erythrocytes.

**Conclusion:**

The ex vivo model provides a novel way of studying receptor-ligand interactions and antibody mediated inhibition of binding in placental malaria.

**Electronic supplementary material:**

The online version of this article (doi:10.1186/s12936-016-1342-2) contains supplementary material, which is available to authorized users.

## Background

Pregnancy-associated malaria is a major cause of morbidity among pregnant women and their offspring in *Plasmodium falciparum* endemic areas. *Plasmodium falciparum* infected erythrocytes sequester in the intervillous space, causing placental malaria. Pregnancy-associated malaria is associated with placental intervillositis, maternal anemia and low birth-weight [1]. Current measures to protect pregnant women from pregnancy-associated malaria are insecticide-treated bed nets, intermittent preventive treatment in pregnancy and treatment of infections [2]. However, pregnancy-associated malaria is often asymptomatic and may occur before the first antenatal visit [3]. Increasing resistance to anti-parasite and anti-mosquito drugs along with changed vector behaviour is reducing efficacy of current protective measure for pregnant women. Parasites express *P. falciparum* erythrocyte membrane protein 1 (PfEMP1) on the surface of infected erythrocytes, mediating cytoadhesion to endothelial cells, platelets, erythrocytes and syncytiotrophoblast, thereby evading circulation and destruction in the spleen. VAR2CSA, a unique member of the PfEMP1 protein family, was discovered in 2003 [4], since then a large base of evidence supports the causal relationship between VAR2CSA and placental malaria [5–12]. Parasites infecting pregnant women bind to chondroitin sulfate A (CSA) [13] and recombinant VAR2CSA bind with high affinity to CSA [14–16]. However, binding to immunoglobulin and hyaluronic acid have also been associated with placental malaria [17–19]. Furthermore, it is not known whether parasites binding to receptors other than CSA can accumulate in the placenta as such parasites are restricted by immunity, since women in malaria endemic regions develop protective antibodies during childhood. Interaction with multiple receptors may have implications for how the pathology manifests during infections, but also for the development of a vaccine to induce antibodies that inhibit the binding of infected erythrocytes to placental tissue. This is an important question in areas of reduced malaria prevalence, as less exposure to malaria in childhood may affect development of protective immunity, leaving women more susceptible to infection when they reach reproductive age. Currently, adhesion blocking capacity of antibodies has largely been tested in assays where only one receptor, namely CSA, is present [20–22], however the efficacy of such antibodies may be limited if sequestration occurs by other pathways. Recent work have shown that human placental and cancer cells express a distinct form of chondroitin sulfate, that is not present in other normal human tissue [23]. Interpretation of binding assays using bovine CSA is, therefore, a major concern, as VAR2CSA-expressing infected erythrocytes are likely to bind with higher affinity to placental CSA. Studies of the mechanisms of placental sequestration have used placental tissue cryosections, however, these studies are contradictory, as they have demonstrated both exclusive CSA dependence and involvement of immunoglobulin binding [18, 24, 25]. Some of these differences may have been incurred by the fixation of tissues that can damage secondary protein structure resulting in alteration of important epitopes and/or receptors.

There are established models in which adhesion of infected erythrocytes is studied under homogenous flow conditions [20, 24]. Although important knowledge of parasite adhesion can be derived from these models, they do not simulate the complex flow through the villous tree in the intervillous space. Modelling of the intervillous blood flow suggests that there is a pressure gradient depending on the spacing between spiral artery inflow and venous outflow, vessel caliber, and maternal blood flow rate [26].

Establishment of in vivo murine models is difficult as murine malaria parasites do not possess PfEMP1 s [27] or knob structures [28] although it has previously been shown that *Plasmodium berghei* infected erythrocytes accumulate in the placental tissue of BALB/c mice due to low blood flow [29]. Furthermore, a *var2csa* transgenic *P. berghei* murine placental model has recently been developed [30]. *Plasmodium falciparum* infection models in *Aotus* monkeys are currently being developed but they are also associated with considerable costs and difficulties. Buffet et al. described an ex vivo perfusion model utilising human spleen to investigate clearance of drug treated *P. falciparum* infected erythrocytes [31]. In this paper, adaptation of a human placental perfusion model that replicates natural infected erythrocyte binding to intact human placental tissue under flow conditions close to the physiological placental flow is described. The dual ex vivo placental perfusion model, where the maternal and fetal circulation is re-established in a human placental cotyledon, has been used extensivley for many years to study placental physiology, such as transfer of nutrients and IgG across the placental barrier [32, 33]. Placental perfusion has shown the role of IgG in mediating merozoite surface protein-1 antigen transfer from mother to fetus [34]. The model has also been used to study placental infections with enterovirus [35] and cytomegalovirus [36].

In this study, the modification of the placental perfusion model to study adhesion of malaria parasite-infected erythrocytes is described. The model may provide new insights into the receptor specificity and availability for binding of *P. falciparum* infected erythrocytes in placental tissue.

## Methods

### Parasite cultures

Parasite isolates were cultured in vitro as previously described [7, 37]. Briefly, parasites were maintained in flasks at 5 % haematocrit of human blood group O+ in RPMI 1640 HEPES medium supplemented with 25 mM NaHCO_3_, 4 mM l-glutamine, 0.125 g/l Gentamicin (all Sigma-Aldrich), 0.125 g/L Albumax II (Gibco) and 2 % human serum. Parasite cultures of the FCR3 and NF54 genotypes were selected for binding to BeWo cells [38, 39], which resulted in a parasite culture that expressed exclusively VAR2CSA [7] and FCR3 parasite cultures were selected for binding to recombinant Endothelial Protein C Receptor (EPCR), which resulted in IT4VAR19 PfEMP1 expression [40]. A transgenic parasite, DC-J, in which a PfEMP1 coding region was replaced with a drug selectable marker silencing *var* genes, was used as a negative control [41]. PfEMP1 expression in transgenic parasites were silenced by addition of 2 µg/ml Blasticidin (Gibco). Cultures were kept synchronous by repeated enrichment for late stage infected erythrocytes by exposure to a strong magnetic field [42]. Cultures were assessed for viability, stage, and parasitaemia by Giemsa-stained smears, washed and used in the perfusion experiments immediately after preparation or stored at +4 °C until used in later phases to prevent schizonts from rupturing (up to 9 h).

Parasite cultures were routinely analysed for Mycoplasma infection and genetic identity of the parasite lines was regularly verified by PCR [43, 44].

### Ex vivo placental perfusion

**Fig. 1:**
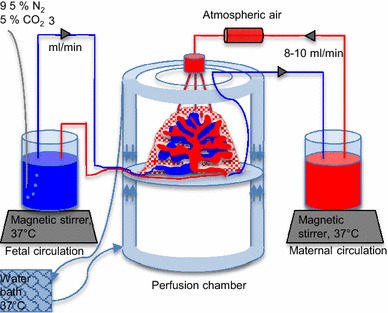
Ex vivo placental perfusion model. After cannulation of a fetal artery and vein pair, the cotyledon is placed in the perfusion chamber with the maternal side facing up. Access to the intervillous space is provided by penetration of the decidual plate with three blunt cannulas. The maternal perfusate leaves the intervillous space through venous openings and is returned to the maternal circulation through a tube connected to a peristaltic pump. The perfusion chamber is surrounded by a water jacket connected to a 37 °C water circuit. The maternal and fetal reservoirs are placed on hot magnetic stirrers to ensure a temperature of the perfusate of 37 °C. The fetal perfusate is equilibrated with 95 % N_2_ and 5 % CO_2_ which is added to the reservoir. An oxygenator inserted into the maternal circulation equilibrates the maternal perfusate with atmospheric air

The perfusion model was adapted from the models described and validated by Schneider et al. [45] and Mathiesen et al. [46] and the work was initiated by the Greifswald perfusion team [47]. Placentas were donated by healthy pregnant women undergoing elective caesarean section at term at Copenhagen University Hospital, Denmark. Placentas were obtained immediately after delivery and Krebs–Ringer buffer with 10 mM glucose and 1:9 citrate (Sigma-Aldrich) was injected into the fetal arteries. A chorionic artery and vein pair was cannulated with neonatal feeding tubes (Flocare) and Krebs–Ringer buffer was infused. The perfused cotyledon and surrounding tissue was placed on a holding ring and fixed with a clamp. The cotyledon was placed in a heated chamber and the fetal and maternal systems were connected to peristaltic roller pumps (Watson Marlow SciQ 323E/D) to create a flow rate of 3–4 ml/min in the fetal circulation and 8–10 ml/min in the maternal circulation (Fig. 1). The maternal circulation was established by introduction of three blunted cannulas through the basal plate into the intervillous space and venous return was continuously drained to the maternal reservoir. Before recirculating the fetal and maternal media an open-loop wash-period was performed. The intervillous space was flushed with 300 ml of RPMI 1640 with gentamicin 0.125 g/l. The media in both circulations were then changed and closed-loop perfusion was started. The fetal medium was equilibrated with 5 % CO_2_, 95 % N_2_. The perfusions were performed without addition of gas to the maternal medium or with insertion of an oxygenator (living systems instrumentation) in the maternal circuit to equilibrate the maternal perfusate with atmospheric air.

Oxygen tension, pH, glucose consumption and lactate production was analysed every 30 min using an ABL90 Flex (Radiometer, Denmark). The fetal and maternal perfusion medium (100 ml) consisted of RPMI 1640 HEPES medium supplemented with 0.125 g/l Gentamicin, human serum albumin (CSL Behring) or bovine serum albumin (Saveen & Werner AB) 40 mg/ml in the fetal medium and 30 mg/ml in the maternal medium [46], and either uninfected erythrocytes (human blood group O+) or late stage *P. falciparum* infected erythrocytes at a haematocrit of 1 %. A physiological haematocrit was not used due to technical difficulties and challenges of culturing parasites in large amounts. Before the experimental phases, a 60 min closed-loop stabilization phase with uninfected erythrocytes in both circulations ensured that the tissue was intact and viable. Shunts between the fetal and maternal circulation were detected as a decreasing fetal output flow rate >0.1 ml/min and resulted in termination of the experiment.

### Experimental design

To examine whether (1) VAR2CSA expressing parasites bind to CSA in an exclusive manner in the placenta and (2) whether parasites associated with severe childhood malaria (EPCR-binding) accumulate in placental tissue, the following experiments were designed:

*A: accumulation of VAR2CSA expressing, EPCR binding and PfEMP1 knockdown parasite in placental tissue:* The maternal medium was substituted with fresh perfusion medium containing late stage erythrocytes infected with FCR3-CSA, FCR3-IT4VAR19 (EPCR binding), NF54-CSA or the transgenic DC-J parasite (PfEMP1 knockdown). After 60 min closed-loop perfusion the experiment was terminated.

*B: binding inhibition with soluble CSA:* The maternal medium was substituted with medium containing late stage FCR3-CSA expressing erythrocytes pre-incubated for 10 min with 800 µg/ml soluble bovine CSA (Fluka). After 60 min closed-loop perfusion (Phase 1), the maternal circulation was washed to flush out infected erythrocytes. The maternal medium was changed to fresh medium with late stage FCR3-CSA expressing erythrocytes and another closed perfusion was performed for 60 min (Phase 2).

*C: binding inhibition with serum:* late stage FCR3-CSA infected erythrocytes were preincubated for 30 min with serum from rabbits immunized with full-length VAR2CSA (FV2). Pre-incubation of 1 ml parasite culture resuspended in RPMI at a total volume of 10 ml was performed at serum concentrations 1:1000, 1:100, and 1:10. After pre-incubation albumin and RPMI were added to achieve 100 ml perfusion medium. Perfusion experiments were performed in four phases separated by a wash phase to flush out unbound infected erythrocytes and serum; Phase 1: FCR3-CSA without serum, Phase 2: FCR3-CSA with anti-FV2 serum 1:1000, Phase 3: FCR3-CSA with anti-FV2 serum 1:100, Phase 4: FCR3-CSA with anti-FV2 serum 1:10 (n = 2) and in the reversed order (n = 3).

During experimental phases, maternal perfusate samples were collected from the maternal reservoir at 10 min intervals.

### Protein production

The recombinant full-length VAR2CSA antigen was produced essentially as described [48]. Briefly, *var2csa* were made as expression-optimized genes at GeneArt (LifeTechnologies). Gene fragments were cloned into the *baculovirus* vector pAcGP67-A (BD Biosciences) and used to transfect High five (Life technologies) cells. The full length baculo produced protein was purified on Ni2+ -sepharose using an ÄKTA-express purification system followed by size exclusion. All proteins were quality controlled by reduced and non-reduced SDS page, ELISA and western blot [49, 50].

### Immunizations

Full-length VAR2CSA antisera was produced in a New Zealand white rabbit by subcutaneous injection of 80 µg recombinant VAR2CSA in Freund’s complete adjuvant, followed by four booster injections of 40 µg of protein in Freund’s incomplete adjuvant. Antisera were collected 14 days after the final boosting injection.

### Flow cytometry

The proportion of infected erythrocytes in the maternal circulation was determined by flow cytometry. Perfusate was diluted 1:20 in RPMI and stored at 4 °C until analysed (within 2–10 h). Parasites were labelled with ethidium bromide (2 µg/ml) in 100 µl aliquots, incubated for 10 min, and washed twice with 200 µl PBS with 2 % FCS. Data were acquired using an FC500 flow cytometer (Beckman Coulter). All samples from one perfusion were processed and analysed together. Data were analysed using FlowJo 7.6 by gating on the ethidium bromide positive cell population and recording the percentage of cells relative to the total cell count [51].

### Histology

A full-thickness placental biopsy from the perfused cotyledon, 1 × 1 × 1 cm, was fixed in 10 % buffered formalin, paraffin embedded, sectioned, mounted on slides and haematoxylin-eosin stained. Sections were examined by light microscopy at magnification ×60. Infected erythrocytes were identified as erythrocytes with coarse brown granular material, birefringent in polarized light. On each slide, intervillous erythrocytes were counted in 50 fields and classified as uninfected erythrocytes, infected erythrocytes in the intervillous space, or infected erythrocytes adjacent to the syncytiotrophoblast. Variation in erythrocyte density in the intervillous space within a section may reflect variation in circulation dynamics during the perfusion. To account for this, parasite density was calculated as a proportion or per 500 uninfected erythrocytes.

### Transmission electron microscopy

Samples from perfused tissue (3 × 3 × 3 mm) were fixed for 2 h at room temperature in fixative [1.5 % paraformaldehyde (TAAB) and 1.5 % glutaraldehyde (Sigma-Aldrich) in 0.1 M Sørensen buffer (pH 7.2)], washed in 0.1 M Sørensen buffer and stored overnight at 4 °C in 0.3 M Sørensen buffer. The tissue was immersed in 1 % osmium tetroxide for secondary fixation. Samples were dehydrated, embedded in Poly/Bed 812^®^ (Polysciences), and 50 nm sections were prepared for ultrathin sectioning on a Leica EM UC7 ultramicrotome. Sections were stained with 4 % uranyl acetate and 1 % lead citrate. Transmission electron microscopy was performed using a Tecnai Spirit Biotwin (FEI Company) microscope.

### Scanning electron microscopy

Samples from perfused tissue were excised and fixed as described above. After primary fixation, samples were dehydrated in increasing concentration of ethanol, critical point dried (Baltzer CPD030), mounted on stubs, sputter coated with chromium (Quorum 150T ES), and visualized in a Jeol JSM-7800F at 1 kV.

### Ethics approval and consent to participate

All study participants gave informed written consent before donation of their placenta. The study was approved by the ethical review board in Capital Region of Denmark (reference nr H-1-2012-103). Animal experiments complied with national and international rules for vaccination, handling, daily care and welfare. Animals were euthanized with fentanyl citrate and fluanisone prior to blood sampling and sacrificed with Phenobarbital. The study was approved by the Danish Animal Experiments Inspectorate (approval number: 2013-15-2934-00902/BES). The methods were carried out in accordance with the approved guidelines for human subjects and experimental animals.

## Results

A series of 15 perfusions were performed and several steps were taken to optimize the model for perfusion with *P. falciparum* infected erythrocytes (Fig. 1).

### Oxygenation

During implementation of the perfusion model the maternal perfusate was oxygenated with 20 % CO_2_, 80 % O_2_, as previously described [52], which led to a supra-physiological oxygen tension and hemolysis. By refraining from oxygenating the maternal perfusate, experiments with uninfected and infected erythrocytes could be performed. The oxygen tension was approximately 20–21 kPa after preparation of the perfusate and decreased during perfusion as oxygen was consumed by the placenta. However, during the 1-h experimental phases, maternal oxygen tension rarely fell below that of arterial blood. Subsequently, an oxygenator (Living Systems Instrumentation) was inserted into the maternal circulation to equilibrate the maternal perfusate with air, which led to more stable oxygen tension. Thus, the perfusions in the dataset below were performed either without oxygenation of the maternal perfusate or using an oxygenator (Additional file 1).

### Coagulation

Heparin has been used during ex vivo placental perfusion to avoid coagulation before cannulation. However, heparin may inhibit the cytoadherence of *P. falciparum* infected erythrocytes [53], therefore, Krebs–Ringer buffer with citrate was injected into the fetal arteries immediately after delivery, and during cannulation and wash of the fetal circulation. No anticoagulant was used after the wash phase or in the maternal circulation. Initially, the volume of the maternal washing medium was 100 ml. In these perfusions, remaining maternal blood and clotted maternal cannulas were observed. Increasing the volume of the washing medium to 300 ml prevented coagulation.

### Cryopreserved versus fresh in vitro parasite cultures

For practical reasons, it would be an advantage to use cryopreserved late stage parasites [54] for perfusion. However, it was technically challenging to thaw the large volume of parasites needed within the timeframe of the perfusion experiment and to maintain the integrity of a high percentage of infected erythrocytes. Thus, for the experiments reported here, live cultured parasites were used. The use of live cultures complicates the model, as the placental perfusion must be performed in close proximity to a laboratory that can maintain high quality parasite cultures.

### Quality parameters

The integrity of the fetal circulation is often assessed by monitoring fetal flow rate and loss of fetal perfusate. In this study, the focus was on the maternal circulation and leakage did not affect parasite adhesion. Caution may be necessary in the assessment of fetal flow rate and loss of fetal perfusate as variation in flow rate between phases was observed, as a result of different colloid osmotic pressure in Krebs–Ringer and medium with or without albumin.

After implementation and optimization of the model, 20 perfusions were performed to test the binding of infected erythrocytes. Quality parameters and viability characteristics during ex vivo perfusions are shown in Additional file 1.

### VAR2CSA expressing *P. falciparum* infected erythrocytes accumulate in ex vivo perfused placental tissue

**Fig. 2:**
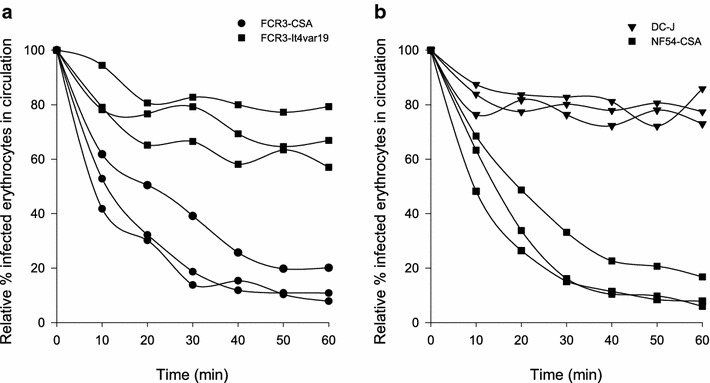
Accumulation of *Plasmodium falciparum* infected erythrocytes in ex vivo perfused placental tissue. The *figure* shows the proportion of infected erythrocytes in the perfusate as a function of time. Results from twelve individual perfusion experiments are shown [FCR3-CSA (n = 3), FCR3-It4var19 (n = 3), NF54-CSA (n = 3) and DC-J (n = 3)]. The number of infected erythrocytes in the maternal perfusate is expressed as relative % of t = 0. **a** FCR3 parasites expressing VAR2CSA disappear from the circulation while FCR3-It4var19 remain in the maternal circulation. **b** NF54 parasites expressing VAR2CSA disappear from the circulation while NF54 parasites that have been silenced for PfEMP1 expression remain in the circulation

FCR3 and NF54 parasites were repeatedly selected for VAR2CSA expression by binding to BeWo cells. To test whether the expression of VAR2CSA enabled binding in the placenta, infected erythrocytes were perfused through placental tissue. Flow cytometry of perfusate samples from the maternal circulation showed that FCR3-CSA and NF54-CSA infected erythrocytes disappeared from the maternal circulation, indicating that they accumulated in the perfused tissue. By contrast, FCR3-IT4VAR19 (EPCR binding) or DC-J (PfEMP1 silenced) infected erythrocytes remained in circulation (Fig. 2). The majority of the VAR2CSA expressing parasites disappeared from the maternal circulation within 30 min and the proportion of infected erythrocytes in the perfusate reached a plateau at approximately 15 % of the infected erythrocytes injected at t = 0. There were no binding characteristics differences between parasite cultures that were used in the perfusion experiment immediately after preparation or stored at +4 °C to prevent rupture of schizonts until used in later phases (Additional file 2).

**Fig. 3:**
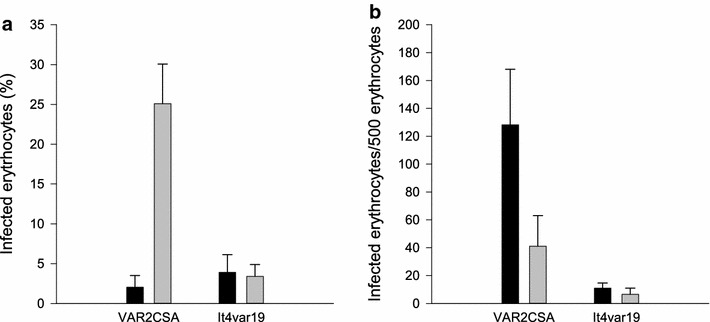
Accumulation of infected erythrocytes in placental tissue. **a** The *bars* indicate the percentage of infected erythrocytes in the perfusate at t = 0 (*black bar*) and the percentage of infected erythrocytes in the intervillous space in sections of perfused tissue (*grey bar*). **b** The *figure* shows the distribution of infected erythrocytes within the perfused tissue, in the intervillous space (*black bars*) or adjacent to the syncytiotrophoblast (*grey bars*). The number of infected erythrocytes is expressed relative to 500 uninfected erythrocytes. Shown are results from three individual FCR3-CSA perfusions and three FCR3-It4var19 perfusions. *Bars* represent mean and standard deviation

**Fig. 4:**
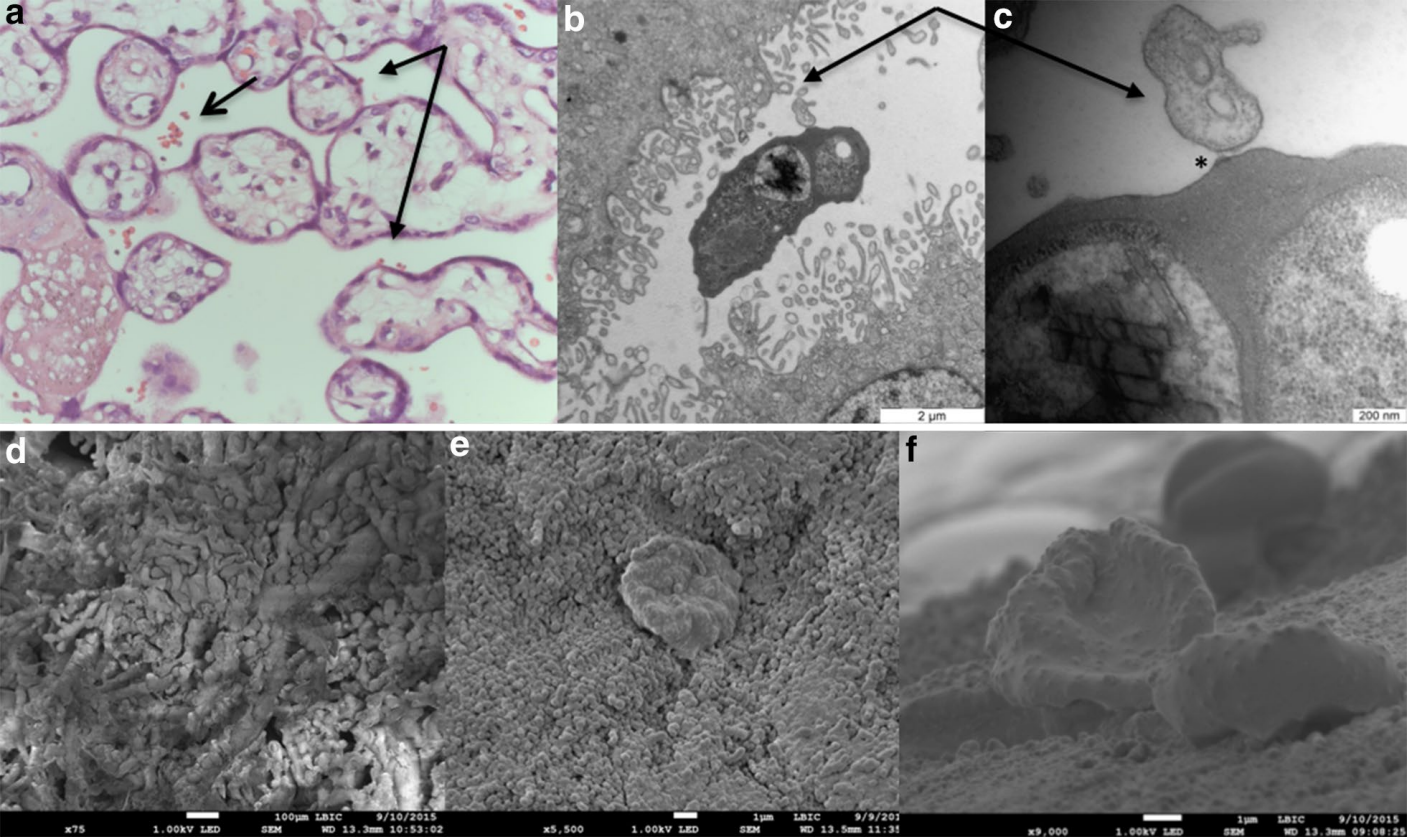
Adhesion of *Plasmodium falciparum* infected erythrocytes in ex vivo perfused placental tissue. **a** Haematoxylin eosin stained section of placental tissue perfused with FCR3-CSA (magnification × 60). Infected erythrocyte accumulation in the intervillous space (*open arrow*) and on the syncytiotrophoblast (*closed arrows*) is shown. **b** Transmission electron micrograph of an FCR3-CSA infected erythrocyte in the intervillous space adjacent to syncytiotrophoblast microvilli (*arrow*) on both sides. **c** Magnification of **b** showing filamentous material connecting a knob on the infected erythrocyte (*star*) and a syncytiotrophoblast microvillus (*arrow*). **d** Scanning electron micrograph of the villous tree showing the complex architecture through which the maternal blood flows. **e**, **f**. Scanning electron micrographs showing VAR2CSA expressing infected erythrocytes adhering to syncytiotrophoblast

Accumulation of infected erythrocytes in perfused tissue was confirmed with light microscopy of haematoxylin-eosin stained sections of placental tissue perfused with infected erythrocytes. In tissue perfused with VAR2CSA expressing parasites, the proportion of erythrocytes that were infected increased after 60 min perfusion. There was no increase in the proportion of infected erythrocytes in tissue perfused with FCR3-It4var19 infected erythrocytes (Fig. 3a). A large proportion of ex vivo sequestered VAR2CSA expressing infected erythrocytes accumulated in the intervillous space rather than on the syncytiotrophoblast (Figs. 3b and 4a).

Transmission electron microscopy of FCR3-CSA perfused tissue showed interaction between placental syncytiotrophoblast microvilli and electron dense knobs on infected erythrocytes (Fig. 4b–c). Electron dense filaments were seen connecting infected erythrocytes and syncytiotrophoblast microvilli. Scanning electron microscopy of FCR3-CSA perfused tissue showed infected erythrocytes adhering to the syncytiotrophoblast surface (Fig. 4 e–f).

### Accumulation of infected erythrocytes is inhibited by soluble CSA and VAR2CSA specific antibodies

**Fig. 5:**
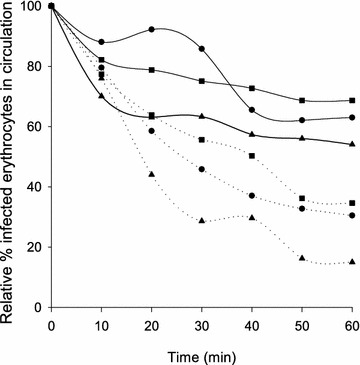
Soluble CSA inhibit FCR3-CSA infected erythrocyte acculmulation in perfused tissue. The Figure shows the proportion of infected erythrocytes in the perfusate as a function of time. Three individual perfusion experiments (indicated by *circle*, *square* or *triangle*) with FCR3-CSA incubated with soluble bovine CSA in Phase 1 (*solid line*) and FCR3-CSA in Phase 2 (*dotted line*). Soluble bovine CSA partly inhibits accumulation of FCR3-CSA in perfused placental tissue

Pre-incubation of VAR2CSA expressing parasites with soluble bovine CSA did not block accumulation of infected erythrocytes even at high concentration, but accumulation was inhibited approximately 50 %, indicating that placental sequestration of VAR2CSA expressing parasites was CSA dependent (Fig. 5).

**Fig. 6:**
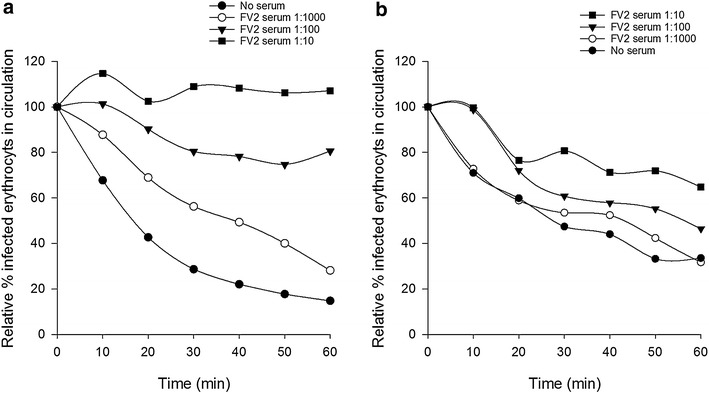
VAR2CSA specific antibodies inhibit FCR3-CSA infected erythrocyte acculmulation in perfused tissue. **a** Serum from a rabbit immunized with the full-length VAR2CSA protein in increasing concentrations inhibits binding of VAR2CSA expressing infected erythrocytes in perfused tissue (representative of two experiments). **b** Serum against FV2 as in **a** but in decreasing concentration (representative of three experiments)

To evaluate the capacity of the perfusion model to measure antibody mediated inhibition of binding, infected erythrocytes were pre-incubated with anti-VAR2CSA antibodies against the full length VAR2CSA. A concentration dependent inhibition of binding was observed with FV2 specific serum (Fig. 6a–b).

## Discussion

The existing knowledge of parasite sequestration in placental malaria originates from field studies and laboratory models showing that parasites from pregnant women bind to CSA [13], that sera from pregnant women previously exposed to placental malaria inhibit parasite binding to CSA [55], that anti-VAR2CSA antibodies are predominantly acquired by women exposed to malaria during pregnancy and rarely found at high concentration in malaria exposed men or children. Moreover, high levels of anti-VAR2CSA IgG in pregnant women are associated with favourable birth outcomes [8]. The occurrence of placental malaria in pregnant women in malaria-endemic areas that have developed protective antibodies towards a wide array of blood stage antigens during childhood underlines the importance of acquiring antibodies, most likely anti-adhesive, towards PfEMP1 molecules. Furthermore, binding to receptors other than CSA has been associated with placental malaria [17–19]. This could have important implications for the etiology of pathology and for the development of vaccines that aim to reduce parasite accumulation in placental tissue. Therefore, an ex vivo placental perfusion model was implemented to study parasite adhesion in intact placental tissue.

Infected erythrocytes expressing VAR2CSA, of both the FCR3 and NF54 genotype, accumulated in the perfused tissue, whereas an EPCR binding (FCR3-IT4VAR19) and a transgenic parasite where PfEMP1 expression had been silenced (DC-J) did not accumulate in the placenta. There was a small loss of It4var19 and DC-J infected erythrocytes, which may be explained by late stage erythrocyte rupture during the experiment.

These results show that the ex vivo perfusion model is suitable to measure placental accumulation of parasites and confirm that infected erythrocyte accumulation in placental tissue is VAR2CSA specific. Furthermore, as the EPCR-binding parasite did not accumulate in placental tissue, in spite of expression of EPCR in human placenta [56] and on the surface of the syncytiotrophoblast [57], it is likely that infected erythrocytes binding to receptors other that CSA will not accumulate in placental tissue. The exclusive interaction with CSA is possibly conferred by shielding of other receptors by the glycocalyx [58]. This suggest a minor role for binding to immunoglobulin and hyaluronic acid [19, 59, 60], but further studies elucidating the involvement of these receptors in the placental perfusion model could shed further light on these observations. Taken together with the finding that disruption of the *var2csa* gene impairs adherence to syncytiotrophoblast in vitro [11] the findings in this study imply that a vaccine against placental malaria can be limited to target only the CSA binding epitope of VAR2CSA.

An advantage of the perfusion model is the opportunity to study parasite adhesion to intact placental tissue under physiologically relevant flow conditions. The details of the flow dynamics of a placental cotyledon still remain to be elucidated, but different methods can be used to estimate the intervillous flow (see Additional file 3 for a discussion). An intervillous flow of 8–10 ml/min divided on three cannulas to optimize the oxygen delivery to the cotyledon is estimated to be within the physiological range. Methodological studies investigating the effect of increasing the number of maternal cannulas and the flow rate during ex vivo placental perfusion have been performed [61, 62]. Increased spiral artery velocity may impair maternal-fetal exchange and damage the villous tissue [63] and was associated with morphological damage of the syncytiotrophoblast in the perfusion model [62]. Although replicating the physiological flow in the perfusion model is a challenge, a flow is created where the maternal perfusate percolates the villous tree at a pressure gradient from the maternal cannulas to the outflow through the basal plate thus simulating the intervillous blood flow. This is an advantage compared to placental malaria models using a capillary or chamber flow system without the villous tree.

Another advantage of the perfusion model is that the intervillous space of the placenta is intact and unfixed so that adherence of infected erythrocytes, not only to the syncytiotrophoblast, but also in the intervillous space, can be studied. It has been suggested that infected erythrocytes bind to a loose matrix of chondroitin sulphate proteoglycan chains in the intervillous space [64]. Histological examination of the perfused tissue showed parasite accumulation both in the intervillous space and to a lesser degree on the syncytiotrophoblast, as seen in women with placental malaria [65]. Relatively few erythrocytes are seen in the fetal vessels and the intervillous space, in perfused tissue compared to non-perfused tissue, as fetal and maternal blood have been washed out [66] and due to the low haematocrit of 1 % in the perfusion experiments. Transmission electron microscopy showed electron dense filaments connecting knobs on the infected erythrocyte with syncytiotrophoblast microvilli as previously shown in tissue from women with placental malaria [67], not limited to denudated areas as recently suggested [68]. Moreover, scanning electron microscopy showed adherence of infected erythrocytes to intact syncytiotrophoblast.

This proof of concept study could form the basis of studying placental pathology in native tissue. However, close collaboration with a labor ward is necessary, as placentas must be accessed immediately after delivery and brought to the laboratory for processing within a short period of time. Moreover, it is imperative that stable and well-characterized PfEMP1 phenotypes are established prior to generating the amounts required for the perfusion experiments. Still, the perfusion group in Greifswald performed pilot experiments, suggesting that the model is indeed transferrable between different centres.

Adhesion of VAR2CSA expressing parasites was inhibited, but not blocked completely by soluble CSA. It has recently been shown that placental chondroitin sulfate shares a specific signature with chondroitin sulfate expressed on cancer cells, which is not found in other healthy human tissues [23]. The incomplete binding inhibition may be caused by differences in parasite tropism for bovine CSA and the human oncofetal CSA found in the placenta.

Antibodies against the full-length VAR2CSA protein inhibited parasite binding in placental tissue in a concentration dependent manner. Some variation was seen between experiments and the binding inhibition appeared greater when the experiments where performed with increasing concentrations of serum compared to the reversed order. One explanation could be that infected erythrocytes that had adhered in the placenta during previous phases were released when a higher concentration of serum was perfused through the tissue. This is a weakness of the model, which needs to be investigated further as it affects the ability to quantify the inhibition capacity if experiments are run in several phases.

## Conclusions

This proof of concept study show that VAR2CSA expressing infected erythrocytes accumulate in the ex vivo placental perfusion model with a distribution similar to that seen in natural placental infection. Parasite accumulation is inhibited by soluble CSA and anti-VAR2CSA antibodies. In conclusion, the perfusion model bestows an opportunity to study parasite binding biology in intact human tissue and the functional properties of antibodies from both pre-clinical and clinical trials.

## Additional files

10.1186/s12936-016-1342-2 Quality and viability characteristics of perfusion experiments.
10.1186/s12936-016-1342-2 Binding of FCR3-CSA infected erythrocytes after 4ºC storage.
10.1186/s12936-016-1342-2 Maternal flow rate during ex vivo placental perfusion.

## Abbreviations

PfEMP1: *P. falciparum* erythrocyte membrane protein 1
CSA: chondroitin sulfate A
EPCR: endothelial protein C receptor
FV2: full-length VAR2CSA

## References

[1] Rogerson SJ, Pollina E, Getachew A, Tadesse E, Lema VM, Molyneux ME (2003). Placental monocyte infiltrates in response to *Plasmodium falciparum* malaria infection and their association with adverse pregnancy outcomes. Am J Trop Med Hyg.

[2] WHO Evidence Review Group (2012). Intermittent preventive treatment of malaria in pregnancy (IPTp) with sulfadoxine-pyrimethamine (SP).

[3] Doritchamou J, Bertin G, Moussiliou A, Bigey P, Viwami F, Ezinmegnon S (2012). First-trimester *Plasmodium falciparum* infections display a typical “placental” phenotype. J Infect Dis.

[4] Salanti A, Staalsoe T, Lavstsen T, Jensen AT, Sowa MP, Arnot DE (2003). Selective upregulation of a single distinctly structured var gene in chondroitin sulphate A-adhering *Plasmodium falciparum* involved in pregnancy-associated malaria. Mol Microbiol.

[5] Duffy MF, Maier AG, Byrne TJ, Marty AJ, Elliott SR, O’Neill MT (2006). VAR2CSA is the principal ligand for chondroitin sulfate A in two allogeneic isolates of *Plasmodium falciparum*. Mol Biochem Parasitol.

[6] Magistrado P, Salanti A, Tuikue Ndam NG, Mwakalinga SB, Resende M, Dahlback M (2008). VAR2CSA expression on the surface of placenta-derived *Plasmodium falciparum*-infected erythrocytes. J Infect Dis.

[7] Nielsen MA, Resende M, Alifrangis M, Turner L, Hviid L, Theander TG (2007). *Plasmodium falciparum*: VAR2CSA expressed during pregnancy-associated malaria is partially resistant to proteolytic cleavage by trypsin. Exp Parasitol.

[8] Salanti A, Dahlback M, Turner L, Nielsen MA, Barfod L, Magistrado P (2004). Evidence for the involvement of VAR2CSA in pregnancy-associated malaria. J Exp Med.

[9] Tuikue Ndam NG, Salanti A, Bertin G, Dahlback M, Fievet N, Turner L (2005). High level of var2csa transcription by *Plasmodium falciparum* isolated from the placenta. J Infect Dis.

[10] Viebig NK, Gamain B, Scheidig C, Lepolard C, Przyborski J, Lanzer M (2005). A single member of the *Plasmodium falciparum* var multigene family determines cytoadhesion to the placental receptor chondroitin sulphate A. EMBO Rep.

[11] Viebig NK, Levin E, Dechavanne S, Rogerson SJ, Gysin J, Smith JD (2007). Disruption of var2csa gene impairs placental malaria associated adhesion phenotype. PLoS One.

[12] Yosaatmadja F, Andrews KT, Duffy MF, Brown GV, Beeson JG, Rogerson SJ (2008). Characterization of VAR2CSA-deficient *Plasmodium falciparum*-infected erythrocytes selected for adhesion to the BeWo placental cell line. Malar J..

[13] Fried M, Duffy PE (1996). Adherence of *Plasmodium falciparum* to chondroitin sulfate A in the human placenta. Science.

[14] Dahlback M, Jorgensen LM, Nielsen MA, Clausen TM, Ditlev SB, Resende M (2011). The chondroitin sulfate A-binding site of the VAR2CSA protein involves multiple *N*-terminal domains. J Biol Chem.

[15] Rieger H, Yoshikawa HY, Quadt K, Nielsen MA, Sanchez CP, Salanti A (2015). Cytoadhesion of *Plasmodium falciparum*-infected erythrocytes to chondroitin-4-sulfate is cooperative and shear enhanced. Blood.

[16] Srivastava A, Gangnard S, Round A, Dechavanne S, Juillerat A, Raynal B (2010). Full-length extracellular region of the var2CSA variant of PfEMP1 is required for specific, high-affinity binding to CSA. Proc Natl Acad Sci USA.

[17] Creasey AM, Staalsoe T, Raza A, Arnot DE, Rowe JA (2003). Nonspecific immunoglobulin M binding and chondroitin sulfate A binding are linked phenotypes of *Plasmodium falciparum* isolates implicated in malaria during pregnancy. Infect Immun.

[18] Flick K, Scholander C, Chen Q, Fernandez V, Pouvelle B, Gysin J (2001). Role of nonimmune IgG bound to PfEMP1 in placental malaria. Science.

[19] Rasti N, Namusoke F, Chene A, Chen Q, Staalsoe T, Klinkert MQ (2006). Nonimmune immunoglobulin binding and multiple adhesion characterize *Plasmodium falciparum*-infected erythrocytes of placental origin. Proc Natl Acad Sci USA.

[20] Fernandez P, Petres S, Mecheri S, Gysin J, Scherf A (2010). Strain-transcendent immune response to recombinant Var2CSA DBL5-epsilon domain block *P. falciparum* adhesion to placenta-derived BeWo cells under flow conditions. PLoS One.

[21] Fried M, Avril M, Chaturvedi R, Fernandez P, Lograsso J, Narum D (2013). Multilaboratory approach to preclinical evaluation of vaccine immunogens for placental malaria. Infect Immun.

[22] Nielsen MA, Pinto VV, Resende M, Dahlback M, Ditlev SB, Theander TG (2009). Induction of adhesion-inhibitory antibodies against placental *Plasmodium falciparum* parasites by using single domains of VAR2CSA. Infect Immun.

[23] Salanti A, Clausen TM, Agerbaek MO, Al Nakouzi N, Dahlback M, Oo HZ (2015). Targeting human cancer by a glycosaminoglycan binding malaria protein. Cancer Cell.

[24] Avril M, Traore B, Costa FT, Lepolard C, Gysin J (2004). Placenta cryosections for study of the adhesion of *Plasmodium falciparum*-infected erythrocytes to chondroitin sulfate A in flow conditions. Microbes Infect.

[25] Muthusamy A, Achur RN, Bhavanandan VP, Fouda GG, Taylor DW, Gowda DC (2004). *Plasmodium falciparum*-infected erythrocytes adhere both in the intervillous space and on the villous surface of human placenta by binding to the low-sulfated chondroitin sulfate proteoglycan receptor. Am J Pathol.

[26] Chernyavsky IL, Jensen OE, Leach L (2010). A mathematical model of intervillous blood flow in the human placentone. Placenta.

[27] Hall N, Karras M, Raine JD, Carlton JM, Kooij TW, Berriman M (2005). A comprehensive survey of the *Plasmodium* life cycle by genomic, transcriptomic, and proteomic analyses. Science.

[28] Mackenstedt U, Brockelman CR, Mehlhorn H, Raether W (1989). Comparative morphology of human and animal malaria parasites I. Host-parasite interface. Parasitol Res.

[29] de Moraes LV, Tadokoro CE, Gomez-Conde I, Olivieri DN, Penha-Goncalves C (2013). Intravital placenta imaging reveals microcirculatory dynamics impact on sequestration and phagocytosis of *Plasmodium*-infected erythrocytes. PLoS Pathog.

[30] de Moraes LV, Dechavanne S, Sousa PM, Barateiro A, Cunha SF, Nunes-Silva S, et al. A murine model for pre-clinical studies on Var2CSA-mediated pathology associated to malaria in pregnancy. Infect Immun. 2016; pii:IAI.01207-15 (Epub ahead of print). 10.1128/IAI.01207-15 PMC4907138 27045035

[31] Buffet PA, Milon G, Brousse V, Correas JM, Dousset B, Couvelard A (2006). Ex vivo perfusion of human spleens maintains clearing and processing functions. Blood.

[32] Mathiesen L, Nielsen LK, Andersen JT, Grevys A, Sandlie I, Michaelsen TE (2013). Maternofetal transplacental transport of recombinant IgG antibodies lacking effector functions. Blood.

[33] Schneider H, Reiber W, Sager R, Malek A (2003). Asymmetrical transport of glucose across the in vitro perfused human placenta. Placenta.

[34] May K, Grube M, Malhotra I, Long CA, Singh S, Mandaliya K (2009). Antibody-dependent transplacental transfer of malaria blood-stage antigen using a human ex vivo placental perfusion model. PLoS One.

[35] Amstey MS, Miller RK, Menegus MA, di Sant’Agnese PA (1988). Enterovirus in pregnant women and the perfused placenta. Am J Obstet Gynecol.

[36] Muhlemann K, Menegus MA, Miller RK (1995). Cytomegalovirus in the perfused human term placenta in vitro. Placenta.

[37] Trager W, Jensen JB (1976). Human malaria parasites in continuous culture. Science.

[38] Haase RN, Megnekou R, Lundquist M, Ofori MF, Hviid L, Staalsoe T (2006). *Plasmodium falciparum* parasites expressing pregnancy-specific variant surface antigens adhere strongly to the choriocarcinoma cell line BeWo. Infect Immun.

[39] Viebig NK, Nunes MC, Scherf A, Gamain B (2006). The human placental derived BeWo cell line: a useful model for selecting *Plasmodium falciparum* CSA-binding parasites. Exp Parasitol.

[40] Turner L, Lavstsen T, Berger SS, Wang CW, Petersen JE, Avril M (2013). Severe malaria is associated with parasite binding to endothelial protein C receptor. Nature.

[41] Dzikowski R, Frank M, Deitsch K (2006). Mutually exclusive expression of virulence genes by malaria parasites is regulated independently of antigen production. PLoS Pathog.

[42] Paul F, Roath S, Melville D, Warhurst DC, Osisanya JO (1981). Separation of malaria-infected erythrocytes from whole blood: use of a selective high-gradient magnetic separation technique. Lancet.

[43] Snounou G, Zhu X, Siripoon N, Jarra W, Thaithong S, Brown KN (1999). Biased distribution of msp1 and msp2 allelic variants in *Plasmodium falciparum* populations in Thailand. Trans R Soc Trop Med Hyg.

[44] Wang CW, Lavstsen T, Bengtsson DC, Magistrado PA, Berger SS, Marquard AM (2012). Evidence for in vitro and in vivo expression of the conserved VAR3 (type 3) *Plasmodium falciparum* erythrocyte membrane protein 1. Malar J..

[45] Schneider H, Panigel M, Dancis J (1972). Transfer across the perfused human placenta of antipyrine, sodium and leucine. Am J Obstet Gynecol.

[46] Mathiesen L, Mose T, Morck TJ, Nielsen JK, Nielsen LK, Maroun LL (2010). Quality assessment of a placental perfusion protocol. Reprod Toxicol.

[47] Cl King, May K, Mostertz J, Paprotka K, Fraunholz M, Grube M (2010). Adherence of *Plasmodium falciparum* infected red cells to the trophoblast and placental inflammatory response studied by dual ex vivo perfusion of an isolated cotyledon. Placenta.

[48] Clausen TM, Christoffersen S, Dahlback M, Langkilde AE, Jensen KE, Resende M (2012). Structural and functional insight into how the *Plasmodium falciparum* VAR2CSA protein mediates binding to chondroitin sulfate A in placental malaria. J Biol Chem.

[49] Nielsen MA, Resende M, de Jongh WA, Ditlev SB, Mordmuller B, Houard S (2015). The influence of sub-unit composition and expression system on the functional antibody response in the development of a VAR2CSA based *Plasmodium falciparum* placental malaria vaccine. PLoS ONE.

[50] Barfod L, Nielsen MA, Turner L, Dahlback M, Jensen AT, Hviid L (2006). Baculovirus-expressed constructs induce immunoglobulin G that recognizes VAR2CSA on *Plasmodium falciparum*-infected erythrocytes. Infect Immun.

[51] Staalsoe T, Giha HA, Dodoo D, Theander TG, Hviid L (1999). Detection of antibodies to variant antigens on *Plasmodium falciparum*-infected erythrocytes by flow cytometry. Cytometry.

[52] Myllynen P, Mathiesen L, Weimer M, Annola K, Immonen E, Karttunen V (2010). Preliminary interlaboratory comparison of the ex vivo dual human placental perfusion system. Reprod Toxicol.

[53] Vogt AM, Barragan A, Chen Q, Kironde F, Spillmann D, Wahlgren M (2003). Heparan sulfate on endothelial cells mediates the binding of *Plasmodium falciparum*-infected erythrocytes via the DBL1alpha domain of PfEMP1. Blood.

[54] Kinyanjui SM, Howard T, Williams TN, Bull PC, Newbold CI, Marsh K (2004). The use of cryopreserved mature trophozoites in assessing antibody recognition of variant surface antigens of *Plasmodium falciparum*-infected erythrocytes. J Immunol Methods.

[55] Fried M, Nosten F, Brockman A, Brabin BJ, Duffy PE (1998). Maternal antibodies block malaria. Nature.

[56] Uhlen M, Fagerberg L, Hallstrom BM, Lindskog C, Oksvold P, Mardinoglu A (2015). Proteomics. tissue-based map of the human proteome. Science.

[57] Faioni EM, Fontana G, Razzari C, Avagliano L, Bulfamante G, Calvi E (2015). Activation of protein C in human trophoblasts in culture and downregulation of trophoblast endothelial protein C receptor by TNF-alpha. Reprod Sci..

[58] Hofmann-Kiefer KF, Chappell D, Knabl J, Frank HG, Martinoff N, Conzen P (2013). Placental syncytiotrophoblast maintains a specific type of glycocalyx at the fetomaternal border: the glycocalyx at the fetomaternal interface in healthy women and patients with HELLP syndrome. Reprod Sci..

[59] Barfod L, Dalgaard MB, Pleman ST, Ofori MF, Pleass RJ, Hviid L (2011). Evasion of immunity to *Plasmodium falciparum* malaria by IgM masking of protective IgG epitopes in infected erythrocyte surface-exposed PfEMP1. Proc Natl Acad Sci USA.

[60] Beeson JG, Rogerson SJ, Cooke BM, Reeder JC, Chai W, Lawson AM (2000). Adhesion of *Plasmodium falciparum*-infected erythrocytes to hyaluronic acid in placental malaria. Nat Med.

[61] Soydemir F, Kuruvilla S, Brown M, Dunn W, Day P, Crocker IP (2011). Adapting in vitro dual perfusion of the human placenta to soluble oxygen tensions associated with normal and pre-eclamptic pregnancy. Lab Invest.

[62] Hutchinson ES, Brownbill P, Jones NW, Abrahams VM, Baker PN, Sibley CP (2009). Utero-placental haemodynamics in the pathogenesis of pre-eclampsia. Placenta.

[63] Burton GJ, Woods AW, Jauniaux E, Kingdom JC (2009). Rheological and physiological consequences of conversion of the maternal spiral arteries for uteroplacental blood flow during human pregnancy. Placenta.

[64] Achur RN, Valiyaveettil M, Alkhalil A, Ockenhouse CF, Gowda DC (2000). Characterization of proteoglycans of human placenta and identification of unique chondroitin sulfate proteoglycans of the intervillous spaces that mediate the adherence of *Plasmodium falciparum*-infected erythrocytes to the placenta. J Biol Chem.

[65] Ismail MR, Ordi J, Menendez C, Ventura PJ, Aponte JJ, Kahigwa E (2000). Placental pathology in malaria: a histological, immunohistochemical, and quantitative study. Hum Pathol.

[66] Maroun LL, Mathiesen L, Hedegaard M, Knudsen LE, Larsen LG (2014). Pathologic evaluation of normal and perfused term placental tissue. Pediatr Dev Pathol.

[67] Bray RS, Sinden RE (1979). The sequestration of *Plasmodium falciparum* infected erythrocytes in the placenta. Trans R Soc Trop Med Hyg.

[68] Hromatka BS, Ngeleza S, Adibi JJ, Niles RK, Tshefu AK, Fisher SJ (2013). Histopathologies, immunolocalization, and a glycan binding screen provide insights into *Plasmodium falciparum* interactions with the human placenta. Biol Reprod.

