# Supplementary material for: Adhesion of Plasmodium falciparum infected erythrocytes in ex vivo perfused placental tissue: a novel model of placental malaria
Source: Malar J. 2016 May 26;15:292. doi: 10.1186/s12936-016-1342-2 (PMC4881162; doi:10.1186/s12936-016-1342-2)
Supplement: Supplementary file 1 — 10.1186/s12936-016-1342-2 Quality and viability characteristics of perfusion experiments. [file 12936_2016_1342_MOESM1_ESM.pdf]

Adhesion of *Plasmodium falciparum* infected erythrocytes in *ex vivo* perfused placental tissue – a novel model of placental malaria

Caroline Pehrson[1*§], Line Mathiesen [2§], Kristine K Heno[1], Ali Salanti[1], Mafalda Resende[1], Ron Dzikowski[3], Peter Damm[4], Stefan R Hansson[5], Christopher L King[6], Henning Schneider[7], Christian W Wang[1], Thomas Lavstsen[1], Thor G Theander[1], Lisbeth E Knudsen[2], Morten A Nielsen[1*]

[1]Centre for Medical Parasitology at Department of Immunology and Microbiology, Faculty of Health and Medical Sciences, University of Copenhagen and at Department of Infectious Diseases, Copenhagen University Hospital (Rigshospitalet), Denmark

[2]Section of Environmental Health, Department of Public Health, University of Copenhagen, Øster Farimagsgade 5A, 1353 Copenhagen, Denmark, Denmark

[3]Department of Microbiology and Molecular Genetics, The Institute for Medical Research Israel - Canada, The Kuvin Center for the Study of Infectious and Tropical Diseases, The Hebrew University-Hadassah Medical School, Jerusalem 91120, Israel

[4]Department of Obstetrics, Rigshospitalet, Faculty of Health and Medical Sciences, University of Copenhagen, Blegdamsvej 9, 2100 Copenhagen Ø, Denmark.

[5]Division of Obstetrics and Gynecology, Department of Clinical Sciences Lund, Lund University, Sweden.

[6]Center for Global Health and Diseases, Case Western Reserve University and Veterans Affairs Medical Center, Cleveland, USA

[7]Department of Obstetrics and Gynecology, Inselspital, Bern University Hospital, University of Bern, Switzerland

[§]These authors contributed equally to this work

[*]Corresponding authors

Caroline Pehrson +45-35332909, cpeh@sund.ku.dk

Morten A Nielsen +45-35326803, mortenn@sund.ku.dk

Additional file 1 - Quality and viability characteristics of perfusion experiments.

**Experiment**	**P211013**	**P141113**	**P091213**	**P171013**	**P211013-2**	**P241013**	**P100314[a]**	**P170314[a]**	**P200314[a]**	**P041113**
Maternal age (years)	40	41	32	31			28	50	27	34
Primipara	No	Yes	Yes	No			No	No	Yes	No
Newborn gender	F	M	F	F	F	M	M	F	M	F
Gestational age (weeks+days)	38+0	39+2	38+2	38+4	38+1	39+2	38+2	39+0	39+1	39+0
Newborn weight (g)	3420	3712	2900	3166	3132	3944	3056	2872	3780	3670
Placenta weight (g)	706	649	691	818		797	546	695	607	662
Perfused cotyledon weight (g)							19.2	25.3	36.9	
Time from delivery to cannulation of fetal vessels (min)	35	30	27	12		36	31	34	18	40
Volume change fetal circulation (ml/h)	1.7	-1.1	-1.6	1.2	-3.0	-2.2	-1.0	-7.7	-1.4	-4.7
Volume change maternal circulation (ml/h)	-1.3	-1.8	-2.0	2.2	-1.3	-0.3	2.2	3.5	-3.7	2.5
Flow rate fetal circulation (ml/minute)	3.2	3.0	2.8	3.5	3.6	2.9	2.7	3.0	2.7	2.8
pH maternal[b]	7.32±0.08	7.35±0.02	7.25±0.05	7.33±0.12	7.33±0.08	7.39±0.08	7.33±0.06	7.45±0.05	7.37±0.07	7.41±0.03
pH fetal[b]	7.31±0.08	7.41±0.02	7.35±0.09	7.35±0.11	7.36±0.06	7.34±0.03	7.24±0.06	7.38±0.03	7.40±0.02	7.45±0.01
pO_2 maternal (kPa)[b]	17.7±1.0	14.0±2.5	14.1±4.6	14.8±1.5	14.9±3.2	9.5±2.8	19.8±0.8	21.1±0.8		14.2±2.3
pO_2 fetal inflow (kPa)[b]	5.8±5.2	9.7±4.5	4.6±2.8	3.4±2.4	4.4±3.1	6.0±5.7	3.7±1.8	7.6±1.7		7.0±4.2
pO_2 fetal outflow (kPa)[b]	8.0±1.8	8.0±2.5	8.8±4.0	5.8±1.4	6.1±1.1	6.8±1.8	9.2±2.5	6.8±2.5		9.1±2.3
Glucose uptake maternal (µmol/L/min)[c]	39.5±10.1		45.4±11.2	29.0±10.1	47.4±10.4	35.9±7.5	18.3±9.5	14.7±5.7	45.1±10.4	33.8±8.2
Glucose uptake fetal (µmol/L/min)[c]			20.5		25.0±0.8	5.4	7.7±0.8	4.1±0.4	7.8±9.7	3.0±1.9
Lactate release maternal (µmol/L/min)[c]	88.2		102.7±24.5	57.4±9.2	102.5	59.0	47.9±11.8	24.6±25.3	92.2±16.0	74.2±14.4
Lactate release fetal (µmol/L/min)[c]			28.2±7.3	22.6	48.7		16.9±3.7	16.0±7.6	19.8±5.7	-0.1±15.1

**Experiment**	**P111113**	**P181113**	**P030414[a]**	**P070414[a]**	**P240314[a]**	**P270314[a]**	**P310314[a]**	**P220514[a]**	**P260514[a]**	**P170714[a]**
Maternal age (years)	31	31	34	36	35	36	35		40	38
Primipara	No	No	No	No	No	No	No		Yes	No
Newborn gender	F	F	F	M	F	M	M		F	M
Gestational age (weeks+days)	38+0	38+0	39+0	38+4	38+5	39+0	38+2		40+6	38+4
Newborn weight (g)	3068	3774	3274	3634	2826	3800	3752		3970	3980
Placenta weight (g)	608	658	714	968	535	735	692		643	925
Perfused cotyledon weight (g)			17.4	16.4	16.2	7.9	12.4	28.0	24.2	
Time from delivery to cannulation of fetal vessels (min)	28	24	31	32	32	29	28		29	25
Volume change fetal circulation (ml/h)	-1.0	-0.8	-9.7	-1.0	-9.0	-0.4	-4.9	0.7	-3.1	0.1
Volume change maternal circulation (ml/h)	-2.3	-3.3	-3.8	0.4	-0.6	-19	-0.9	-2,0	1.5	-2.6
Flow rate fetal circulation (ml/minute)	3.1	3.1	3.1	3.0	3.1	3.1	3.2	3.5	3.3	3.1
pH maternal[b]	7.37±0.06	7.32±0.07	7.44±0.06	7.38±0.04	7.45±0.05	7.42±0.05	7.36±0.04	7.43±0.07	7.42±0.07	7.35±0.09
pH fetal[b]	7.42±0.03	7.46±0.02	7.33±0.04	7.32±0.05	7.45±0.03	7.47±0.01	7.41±0.02	7.40±0.06	7.44±0.01	7.38±0.01
pO_2 maternal (kPa)[b]	15.0±3.3	15.0±0.8	21.0±0.6					18.8±1.6	18.7±1.5	17.2±2.6
pO_2 fetal inflow (kPa)[b]	8.0±5.1	8.0±4.2	6.4±0.9	9.6±2.6				6.5±2.6	6.3±2.3	5.5±2.1
pO_2 fetal outflow (kPa)[b]	11.5±1.0	9.4±1.7	7.5±1.7				17.9±0.4	7.2±4.0	6.2±1.6	7.6±3.1
Glucose uptake maternal (µmol/L/min)[c]			11.4±5.2	31.7±4.5	19.9±3.3	32.1±10.6	29.2±12.1	50.4±11.1	45.4±11.5	48.0±11.5
Glucose uptake fetal (µmol/L/min)[c]			5.5±5.1	16.4±10.3	3.9±12.1	1.4±5.5	3.4±2.6	12.4±9.9	10.0±7.1	6.8±2.6
Lactate release maternal (µmol/L/min)[c]			33.3±6.7	70.6±11.5	38.4±5.2	69.3±12.6	54.9±24.8	105.2±14.6	94.2±20.4	99.1±16.8
Lactate release fetal (µmol/L/min)[c]			13.7±2.5	9.4±5.1	1.0±11.1	6.9±2.3	7.7±6.7	28.5±13.4	6.7±12.1	10.5±3.2

[a]Experiments performed with an oxygenator equilibrating the medium with atmospheric air inserted in the maternal circulation
[b]Mean ± standard deviation
[c]Mean ± standard deviation of stabilisation and experimental phases (if data was available from one phase only, uptake/release between first and last measurements is shown)
