# Supplementary material for: Adhesion of Plasmodium falciparum infected erythrocytes in ex vivo perfused placental tissue: a novel model of placental malaria
Source: Malar J. 2016 May 26;15:292. doi: 10.1186/s12936-016-1342-2 (PMC4881162; doi:10.1186/s12936-016-1342-2)
Supplement: Supplementary file 2 — 10.1186/s12936-016-1342-2 Binding of FCR3-CSA infected erythrocytes after 4ºC storage. [file 12936_2016_1342_MOESM2_ESM.docx]

# Adhesion of *Plasmodium falciparum* infected erythrocytes in *ex vivo* perfused placental tissue – a novel model of placental malaria

### Caroline Pehrson^1^*^§^, Line Mathiesen ^2§^, Kristine K Heno^1^, Ali Salanti^1^, Mafalda Resende^1^, Ron Dzikowski^3^, Peter Damm^4^, Stefan R Hansson^5^, Christopher L King^6^, Henning Schneider^7^, Christian W Wang^1^, Thomas Lavstsen^1^, Thor G Theander^1^, Lisbeth E Knudsen^2^, Morten A Nielsen^1*^

^1^Centre for Medical Parasitology at Department of Immunology and Microbiology, Faculty of Health and Medical Sciences, University of Copenhagen and at Department of Infectious Diseases, Copenhagen University Hospital (Rigshospitalet), Denmark

^2^Section of Environmental Health, Department of Public Health, University of Copenhagen, Øster Farimagsgade 5A, 1353 Copenhagen, Denmark, Denmark

^3^Department of Microbiology and Molecular Genetics, The Institute for Medical Research Israel - Canada, The Kuvin Center for the Study of Infectious and Tropical Diseases, The Hebrew University-Hadassah Medical School, Jerusalem 91120, Israel

^4^Department of Obstetrics, Rigshospitalet, Faculty of Health and Medical Sciences, University of Copenhagen, Blegdamsvej 9, 2100 Copenhagen Ø, Denmark.

^5^Division of Obstetrics and Gynecology, Department of Clinical Sciences Lund, Lund University, Sweden.

^6^Center for Global Health and Diseases, Case Western Reserve University and Veterans Affairs Medical Center, Cleveland, USA

^7^Department of Obstetrics and Gynecology, Inselspital, Bern University Hospital, University of Bern, Switzerland

^§^These authors contributed equally to this work

^*^Corresponding authors

Caroline Pehrson +45-35332909, [cpeh@sund.ku.dk](mailto:cpeh@sund.ku.dk)

Morten A Nielsen +45-35326803, [mortenn@sund.ku.dk](mailto:mortenn@sund.ku.dk)

Additional File 2. Binding of FCR3-CSA infected erythrocytes after 4º C storage.


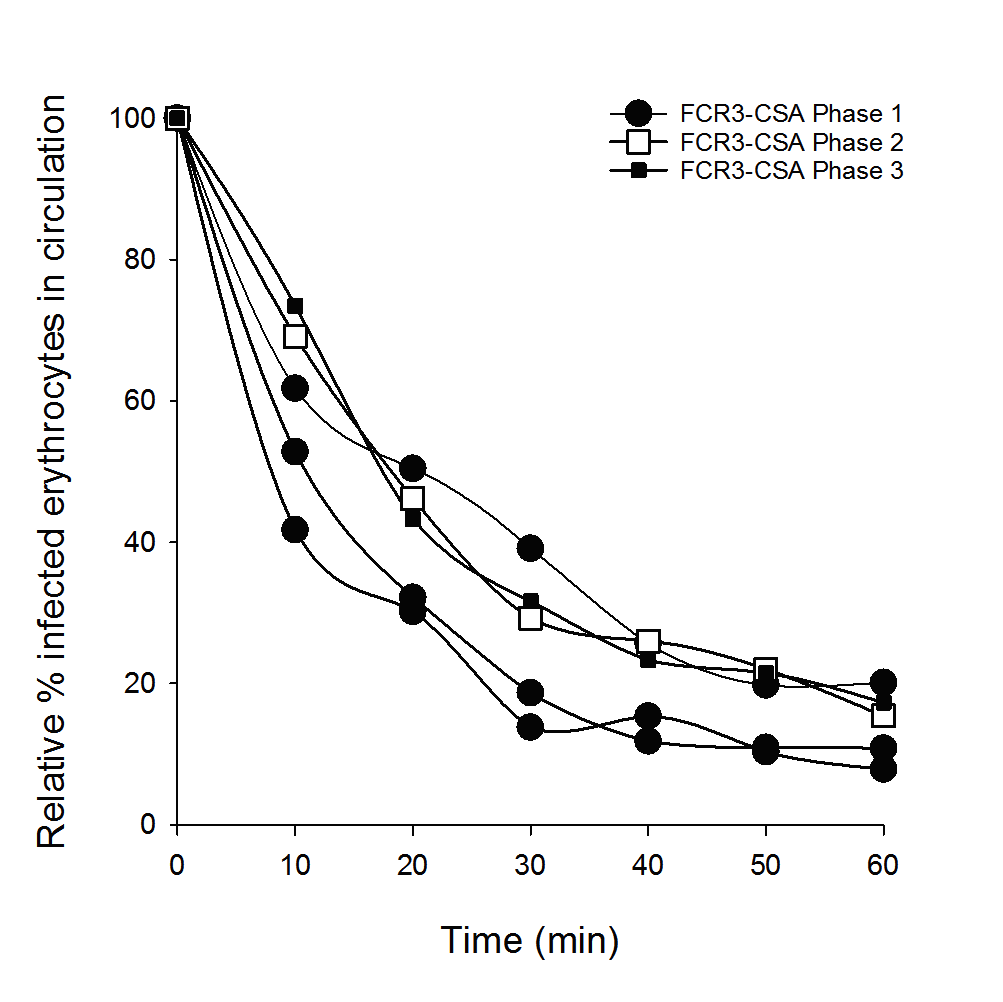


The figure shows three perfusion experiments with FCR3-CSA done on different days (⚫) (also shown in Figure 2). All three experiments were run as phase 1 with maximum 30 minutes storage at +4°C. The figure shows additionally two perfusion experiments with FCR3-CSA (◼ & 🞏) run as perfusion phase 2 or 3, that is, 1.5 or 3 hours after the beginning of phase 1.
