# Supplementary material for: Adhesion of Plasmodium falciparum infected erythrocytes in ex vivo perfused placental tissue: a novel model of placental malaria
Source: Malar J. 2016 May 26;15:292. doi: 10.1186/s12936-016-1342-2 (PMC4881162; doi:10.1186/s12936-016-1342-2)
Supplement: Supplementary file 3 — 10.1186/s12936-016-1342-2 Maternal flow rate during ex vivo placental perfusion. [file 12936_2016_1342_MOESM3_ESM.docx]

**Adhesion of *Plasmodium falciparum* infected erythrocytes in *ex vivo* perfused placental tissue – a novel model of placental malaria**

Caroline Pehrson^1^*^§^, Line Mathiesen ^2§^, Kristine K Heno^1^, Ali Salanti^1^, Mafalda Resende^1^, Ron Dzikowski^3^, Peter Damm^4^, Stefan R Hansson^5^, Christopher L King^6^, Henning Schneider^7^, Christian W Wang^1^, Thomas Lavstsen^1^, Thor G Theander^1^, Lisbeth E Knudsen^2^, Morten A Nielsen^1*^

^1^Centre for Medical Parasitology at Department of Immunology and Microbiology, Faculty of Health and Medical Sciences, University of Copenhagen and at Department of Infectious Diseases, Copenhagen University Hospital (Rigshospitalet), Denmark

^2^Section of Environmental Health, Department of Public Health, University of Copenhagen, Øster Farimagsgade 5A, 1353 Copenhagen, Denmark, Denmark

^3^Department of Microbiology and Molecular Genetics, The Institute for Medical Research Israel - Canada, The Kuvin Center for the Study of Infectious and Tropical Diseases, The Hebrew University-Hadassah Medical School, Jerusalem 91120, Israel

^4^Department of Obstetrics, Rigshospitalet, Faculty of Health and Medical Sciences, University of Copenhagen, Blegdamsvej 9, 2100 Copenhagen Ø, Denmark.

^5^Division of Obstetrics and Gynecology, Department of Clinical Sciences Lund, Lund University, Sweden.

^6^Center for Global Health and Diseases, Case Western Reserve University and Veterans Affairs Medical Center, Cleveland, USA

^7^Department of Obstetrics and Gynecology, Inselspital, Bern University Hospital, University of Bern, Switzerland

^§^These authors contributed equally to this work

^*^Corresponding authors

Caroline Pehrson +45-35332909, [cpeh@sund.ku.dk](mailto:cpeh@sund.ku.dk)

Morten A Nielsen +45-35326803, [mortenn@sund.ku.dk](mailto:mortenn@sund.ku.dk)

# Maternal flow rate during *ex vivo* placental perfusion

The details of the flow dynamics of a placental cotyledon still remains to be elucidated, but different methods have been used to estimate the intervillous flow.

Rekonen et al [1] used an intravenous ^133^Xe method to estimate the intervillous blood flow to 135±49 ml/minute/100 ml intervillous space. Käär et al found [2] similar results in healthy pregnant women in late third trimester (140±53 ml/minute/100ml intervillous space, range 73-261) while the intervillous flow was lower in complicated pregnancies. Assuming a ratio of 1:1 of villi and intervillous space, the intervillous flow/g of tissue can be estimated to approximately 0.7 ml/min in healthy pregnant women close to term suggesting an appropriate perfusion flow of 5.5-25.8 ml/min (perfused cotyledon weight range 7.9-36.9 g, Additional file 1) depending on cotyledon size.

Assuming a uterine arterial blood flow of 750 ml/min [3] and an average placental weight of 703 g (Additional file 1) suggests an appropriate inflow rate of 1.07 ml/g tissue. This results in an intervillous flow between 8.4-39.5 ml/min depending on the size of the cotyledon. This indicates that the flow rate in the perfusion model is within physiological range in smaller cotyledons but low in larger cotyledons. Burton et al [4] estimate the flow in the spiral arteries to 0,2-0,4 ml/s (12-24 ml/min). Both the above estimates are likely to be high as recent findings indicate that part of the blood volume bypass the placenta through subplacental arteriovenous shunts in the myometrium [5]. There is a lack in consensus as to how many spiral arteries supply the placenta, but it is believed that one spiral artery delivers blood into a central cavity of a lobule (reviewed in [6]). Although the flow is divided on three cannulas in this model, the total flow of 10 ml/min to one cotyledon may be appropriate when taking into account that shunting was not included in this estimate.

Although all these estimates are uncertain, we believe the intervillous flow in the model (8-10 ml/min/cotyledon) is within the physiological range.

1. Rekonen A, Luotola H, Pitkanen M, Kuikka J, Pyorala T. Measurement of intervillous and myometrial blood flow by an intravenous 133Xe method. Br J Obstet Gynaecol. 1976; 83:723-8.

2. Kaar K, Jouppila P, Kuikka J, Luotola H, Toivanen J, Rekonen A. Intervillous blood flow in normal and complicated late pregnancy measured by means of an intravenous 133Xe method. Acta Obstet Gynecol Scand. 1980; 59:7-10.

3. Assali NS, Douglass RA, Jr., Baird WW, Nicholson DB, Suyemoto R. Measurement of uterine blood flow and uterine metabolism. IV. Results in normal pregnancy. Am J Obstet Gynecol. 1953; 66:248-53.

4. Burton GJ, Woods AW, Jauniaux E, Kingdom JC. Rheological and physiological consequences of conversion of the maternal spiral arteries for uteroplacental blood flow during human pregnancy. Placenta. 2009; 30:473-82.

5. Schaaps JP, Tsatsaris V, Goffin F, Brichant JF, Delbecque K, Tebache M, et al. Shunting the intervillous space: new concepts in human uteroplacental vascularization. Am J Obstet Gynecol. 2005; 192:323-32.

6. Benirschke K, Burton G, Baergen R. Pathology of the Human Placenta*.* 6th edn. Heidelberg: Springer; 2012.
